# Understanding the Long-Lasting Attraction of Malaria Mosquitoes to Odor Baits

**DOI:** 10.1371/journal.pone.0121533

**Published:** 2015-03-23

**Authors:** Collins K. Mweresa, Bruno Otieno, Philemon Omusula, Berhane T. Weldegergis, Niels O. Verhulst, Marcel Dicke, Joop J. A. van Loon, Willem Takken, Wolfgang R. Mukabana

**Affiliations:** 1 International Centre of Insect Physiology and Ecology, P.O. Box 30772 GPO, Nairobi, Kenya; 2 Laboratory of Entomology, Wageningen University and Research Centre, P.O. Box 8031, EH Wageningen, The Netherlands; 3 School of Biological Sciences, University of Nairobi, P.O. Box 30197 GPO, Nairobi, Kenya; Swedish University of Agricultural Sciences, SWEDEN

## Abstract

The use of odor baits for surveillance and control of malaria mosquitoes requires robust dispensing tools. In this study, the residual activity of a synthetic mosquito attractant blend dispensed from nylon or low density polyethylene (LDPE) sachets was evaluated at weekly intervals for one year without re-impregnation. The potential role of bacteria in modulating the attraction of mosquitoes to odor-treated nylon that had been used repeatedly over the one year study period, without re-impregnation, was also investigated. Significantly higher proportions of female *Anopheles gambiae sensu stricto* mosquitoes were consistently attracted to treated nylon strips than the other treatments, up to one year post-treatment. Additional volatile organic compounds and various bacterial populations were found on the treated nylon strips after one year of repeated use. The most abundant bacteria were *Bacillus thuringiensis* and *Acinetobacter baumannii*. Autoclaving of treated nylon strips prior to exposure had no effect on trap collections of laboratory-reared female *An*. *Gambiae* (P = 0.17) or wild female *An*. *Gambiae* sensu lato (P = 0.26) and *Mansonia* spp. (P = 0.17) mosquitoes. Trap catches of wild female *An*. *Funestus* (P < 0.001) and other anophelines (P < 0.007) were higher when treated strips had been autoclaved prior to deployment as opposed to when the treated nylon strips were not autoclaved. By contrast, wild female *Culex* mosquitoes were more strongly attracted to non-autoclaved compared to autoclaved treated nylon strips (P < 0.042). This study demonstrates the feasibility of using odor baits for sampling and surveillance of malaria as well as other mosquito vectors over prolonged periods of time. Preliminary evidence points towards the potential role of bacteria in sustaining prolonged use of nylon material for dispensing synthetic attractant odorants for host-seeking malaria and other mosquito vectors but further investigations are required.

## Introduction

Prospects of eliminating malaria are largely threatened by changes in the feeding and resting behavior of malaria mosquitoes, emerging resistance of *Plasmodium* parasites and malaria vectors to anti-malarial drugs and insecticides, respectively [1,2]. These obstacles can be partly overcome by developing novel technologies for surveillance and control of malaria mosquitoes [3,4,5]. In the past two decades, various odor-baits and release matrices have been developed and tested for sustained collection of high numbers of insect vectors [6,7]. For example, low density polyethylene (LDPE) has been effectively used for the prolonged release of attractants for tsetse flies and agricultural insect pests [8,9,10]. Although LDPE sachets have also proven suitable for the release of mosquito attractants [11,12,13], recent findings indicate that they attract fewer female *Anopheles gambiae sensu stricto* mosquitoes (hereafter referred to as *An*. *gambiae*) than nylon strips impregnated with similar compounds [14,15]. Furthermore, the treated nylon strips were consistently more attractive to host-seeking *An*. *gambiae* mosquitoes than LDPE sachets containing the same attractants up to 40 nights post-treatment [15]. This suggests that attractant-treated nylon strips possess a residual attraction capacity, which allows for prolonged use. This reduces the time, labour and costs needed for preparing fresh baits. However, it is not known whether original attractants applied on nylon strips can remain intact and active beyond the 40 post-treatment nights reported [15]. Furthermore, based on the reported association between skin microbiota and attraction of *An*. *gambiae* to humans [16,17,18,19,20], it is conceivable that bacteria are likely to be involved in the residual attractive effect of treated nylon on *An*. *gambiae*. This study was designed to (a) investigate the long-lasting attraction of malaria mosquitoes to odor baits, (b) identify whether the original volatile organic compounds (VOCs) impregnated on the nylon strips remained intact up to the end of the experimental period, and (c) assess whether microbes are potentially associated with the long-lasting attraction of malaria mosquitoes to IB1-treated nylon strips.

## Materials and Methods

### Mosquitoes

The Mbita strain of *An*. *gambiae sensu stricto* colony established since January 2001 was reared under ambient climatic conditions (i.e. temperature of 23.2 ± 1.3°C and 77.0 ± 2.6% relative humidity (RH)) and used for semi-field bioassays. All semi-field experiments were conducted within a screen-walled greenhouse at the Thomas Odhiambo Campus of the International Centre of Insect Physiology and Ecology (*icipe-*TOC) (00°25'S, 34°13'E and 1240 m above sea level) located at Mbita Point Township in western Kenya. Adult mosquitoes were blood-fed three times a week on a human arm, and provided with 6% (w/v) glucose solution on filter paper. Eggs were laid on wet filter paper and placed in plastic trays (measuring 35 cm × 25 cm × 5 cm) containing filtered water from Lake Victoria. Large-sized particles were filtered by passing the lake water over wood charcoal to enhance filter-feeding of *An*. *gambiae* mosquito larvae on approximately 0.5 mg of Tetramin baby fish food (Melle, Germany) /larva provided thrice a day. Pupae were collected daily, placed in clean cups (10 cm diameter and 14 cm high) half filled with filtered lake water and then transferred into mesh-covered cages (30 × 30 × 30 cm). A total of 200 female mosquitoes aged 3–5 d and without prior access to a blood meal were randomly aspirated from the cage and starved for 8 h before being used in semi-field experiments (20:00–06:30 h). The mosquitoes were only provided with water on cotton towels placed on top of mosquito holding cups.

### Mosquito attractants

The synthetic mosquito attractant ‘Ifakara blend 1 (IB1)’ [21], combined with carbon dioxide produced by yeast-fermenting sugar [22,23], was used in all experiments. All the eleven constituent compounds of IB1 except carbon dioxide were released from LDPE (Audion Elektro, Weesp, The Netherlands) sachets or strips of nylon (Bata Shoe Company, Kenya). Each LDPE sachet measured 2.5 cm × 2.5 cm and was filled with one milliliter of an individual chemical constituent of IB1. The sheet thicknesses of LDPE sachets used to dispense the individual attractant compounds were 0.2 mm (99.6% propionic acid, 99.9% butanoic acid, 99% pentanoic acid, 99% 3-methylbutanoic acid, and distilled water), 0.1 mm (98% heptanoic acid, 99.9% octanoic acid), 0.03 mm (99% tetradecanoic acid and 25% ammonia solution), and 0.05 mm (L-lactic acid (85%)). Chemicals were purchased from the Sigma-Aldrich Corporation (SIAL: NASDAQ GS, Germany), while distilled water was supplied by Buyimpex Laboratory Equipment Suppliers Limited in Kisumu, Kenya.

Individual nylon strips measuring 26.5 cm × 1 cm were separately soaked in one milliliter of each chemical constituent at optimal concentrations (% v/v) of 0.01% (propionic acid), 1% (butanoic acid), 0.0001% (pentanoic acid, heptanoic acid and octanoic acid), 0.000001% (3-methyl butanoic acid), 0.00025% (tetradecanoic acid), 2.5% (ammonia), 85% (lactic acid) and 100% (distilled water) [14,15]. The nylon strips contained 90% polyamide and 10% spandex. Tetradecanoic and octanoic acids were dissolved in ethanol while propionic, butanoic, pentanoic, heptanoic, 3-methylbutanoic acid and ammonia were dissolved in distilled water. The treated nylon strips were air-dried at room temperature for 5 h before the start of the experiments.

Carbon dioxide (approximately 63 ml/min) was produced by mixing 2 L of tap (semi-field experiments) or river water (field experiments), 17.5 g of instant dry yeast (Angel Company, China) and 250 g of refined sugar (Sony sugar Company Ltd, Kenya) 30 min prior to the start of each experiment [15,21]. This gas was delivered through a silicon tube (0.5 cm internal diameter) into a MM-X trap (American Biophysics, North Kingstown, RI, USA) baited with blend IB1. The LDPE sachets filled with individual attractant chemicals were weighed before and after each experimental night [8,15]. Sachets filled with components were only replaced upon leakage or depletion. The replacement frequency varied significantly among individual components [15,24]. Although control and IB1-treated nylon strips were prepared once and re-used (without replenishment) in all experiments of this study, CO_2_ was prepared anew on each experimental night [15]. Equivalent numbers of 10 untreated LDPE sachets and nylon strips (i.e. controls/without odor bait) [12,23] as well as 10 IB1-treated LDPE sachets and nylon strips were separately suspended on a wire hook and placed within the odor plume tube of the MM-X trap (14).

### Long-lasting attraction of *An*. *gambiae* to treated nylon under semi-field conditions

It has been demonstrated that attractant-impregnated nylon strips lured *An*. *gambiae* mosquitoes for 40 consecutive nights without re-treatment [15]. This follow-up study was aimed at investigating whether this residual effect could be reproduced at weekly intervals over a period of one year (i.e. 52 nights). Four treatments were used: (a) control LDPE sachets (no odor bait), (b) control nylon strips (no odor bait), (c) LDPE sachets filled with blend IB1, and (d) IB1-treated nylon strips. The experiments were randomized by treatment and trap position during each experimental night. Individual treatments were mounted in the odor outlet tube of separate MM-X traps operated on 12 V. The odor outlet end of each trap was suspended at a height of 15 cm above the ground [25,26]. The MM-X traps dispensed odorants and collected mosquitoes attracted to each treatment. Collected mosquitoes did not come into direct contact with the nylon or LDPE sachet materials placed within the trap (14). Treatments were assigned to particular traps throughout the study and alternated nightly to overcome the potential effect of site on mosquito catches [15,21]. Trapping assays for assessment of mosquito attraction to the four treatments were replicated three times per week for one year since impregnation. The mosquitoes collected in individual traps at the end of each experimental night were removed, counted and recorded. Treatments were separately stored in the refrigerator (4°C) between experimental nights. Untrapped mosquitoes were recaptured from the screen-walled greenhouse using manual aspirators and killed. Traps were cleaned using a 70% methanol solution between experiments. The ambient temperature and RH during all experimental nights were recorded using data loggers (Tinytag Ultra, model TGU-1500, INTAB Benelux, The Netherlands).

### Identification of compounds found on nylon strips one year post-treatment

This study was aimed at determining which chemicals were still present on IB1-treated nylon strips that had been repeatedly used to attract *An*. *gambiae* mosquitoes once per week for a period of 52 nights without re-treatment. Headspace sampling was used to collect VOCs released from three sets of strips, each consisting of 10 separate nylon strips (4.5 cm × 1 cm each). Each strip was impregnated with a single component of IB1 only. Volatiles were entrained using a purge and trap on Tenax-TA 20/35 (Markes International, Llantrisant, UK), from each of the ten IB1-impregnated nylon strips put together in separate 5 liter glass containers whose lids were fitted with air inlets and outlets. To reduce background volatiles, air was sucked into the cuvette through a standard glass cartridge containing 200 mg Tenax-TA [19]. Headspace volatiles were entrained at a flow rate of 100 ml/min for two hours on a second cartridge containing 200 mg Tenax-TA connected to the outlet of the cuvette. The samples were analysed using thermal desorption (TD) with gas chromatography and mass spectrometry detection (GC/MS). A Thermo trace GC ultra coupled with Thermo trace DSQ and mass spectrometer (Thermo Fisher Scientific, Waltham, USA) was used for separation and detection of volatile compounds from the nylon strips.

Prior to the release of volatiles, each sample was dry-purged under a flow of nitrogen (20 ml/min) for 10 min at ambient temperature in order to remove moisture. This was followed by release of volatiles thermally from the Tenax TA adsorbent material using ultra 50:50 thermal desorption unit (Markes) at 250°C for 10 min under helium flow of 20 ml/min, while re-collecting the volatiles in a cooled solvent trap at 10°C using UNITY (Markers). Once the desorption process was complete, volatile compounds were released from the cold trap at a fast heating rate of 40°C/s to 280°C for 10 min. The volatiles were transferred to a ZB-5MSi analytical column (30 mL × 0.25 mm I.D. × 1.00 μm F.T. (Phenomenex, Torrance, CA, USA)) in splitless mode for further separation. The GC oven temperature was initially held at 40°C for 2 min and thereafter raised at 10°C/min to a final temperature of 280°C and kept constant for 4 min under a helium flow of 1 ml/min in a constant flow mode. The DSQ mass spectrometer (MS) was operated in scan mode within a range of 35–350 amu at 5.38 scans/s. The spectra were recorded in electron impact ionisation (EI) mode at 70 eV while MS transfer line and ion source were set at 275 and 250°C, respectively. Tentative compound identification was based on comparison of mass spectra with those in the NIST 2005 and Wageningen Mass Spectral Database of Natural Products MS libraries. Experimentally calculated linear retention indices (LRI) were also used as additional measures for confirming the identity of compounds. Average peak areas obtained in the samples were used to estimate the abundance of individual volatiles. Volatiles from the compressed air, empty glass jars, clean Tenax TA adsorbents and the analytical system itself were treated as blank samples and used for correction during the analysis.

### Identification of bacteria found on attractant treated nylon

The purpose of this study was to determine the presence and identity of bacteria found on IB1-treated nylon strips that had been repeatedly used to attract mosquitoes once a week for one year since treatment. All treated nylon strips (4.5 cm × 1 cm) were separately streaked on Trypticase Soy Agar (TSA) plates and incubated overnight at 34°C. The same procedure was repeated by using strips of equal size and number cut from a piece of nylon sock worn for 12 h by a human volunteer and control nylon strips (untreated). The most abundant bacterial species derived from colonies of IB1-treated nylon strips were isolated and identified. Bacterial colonies were picked from the plates using sterile pipette tips and transferred to 20 μl lysis buffer (50 mM NaOH, 0.20% SDS) in 1.5 ml Eppendorf tubes. After heating for 15 min at 95°C, each tube was placed on ice followed by addition of 200 μl water. The samples were centrifuged for 5 min at 12,000 r/min before using 0.5 μl of the supernatant with DNA for Polymerase Chain Reaction (PCR). Before amplification for sequencing, a fingerprinting BOX-PCR [27] was used to confirm that the most abundant colonies on the plate belonged to the same species. Amplification was done by using the universal 16S rDNA primers fD1 and rp2 [28,29]. Amplification products were sequenced using previously described procedures [28,30] (Eurofins MWG Operon, Ebersberg, Germany). The 16S rDNA sequences were compared with those available in the Basic Local Alignment Search Tool (http://blast.ncbi.nlm.nih.gov) to reveal tentative species identity of the bacterial isolates.

### Effect of bacteria-produced volatiles on attraction of *An*. *gambiae* in the laboratory

The attraction of *An*. *gambiae* to volatiles produced by the two most abundant bacterial colonies isolated from IB1-treated nylon strips in the preceding section was evaluated. Both isolates were grown overnight in a liquid medium (15 g tryptone, 5 g soytone and 5 g sodium chloride/1000 ml H_2_O) at 34°C before testing for individual behavioral responses of host-seeking *An*. *gambiae* [19]. The liquid medium was diluted to an optimal concentration of 263cfu/cm^2^ by plating on TSA [19]. Attractiveness of the volatiles produced by the two bacterial isolates to the Suakoko strain of *An*. *gambiae s*.*s* form M. (recently renamed *An*. *colluzzii*) mosquitoes was tested in a dual-port olfactometer [19,31] at the Laboratory of Entomology, Wageningen University, The Netherlands.

For each test, 30 female *An*. *gambiae* mosquitoes aged 5–8 d old which had not received a blood meal, were selected 14 h prior to the experiment and placed in a release cage containing tap water presented on damp cotton wool. The experiments were performed during the last 4 h of the scotophase. In each trial, test odors were released in the air stream before a group of mosquitoes was set free from a cage placed 1.60 m downwind from the two ports. After 15 min, mosquitoes that entered each of the two trapping devices were counted and recorded. Excised blocks of TSA (1.5 × 1.5 × 0.3 cm) with or without bacteria were placed on a glass slide (1.5 × 1.5 cm) and placed in each trapping device [19]. Each treatment was tested four times over two days. The sequence of test odors was randomised on the same day and between days. Test stimuli were alternated between right and left ports of the olfactometer to rule out any positional effects.

### Effect of autoclaving on attraction of *An*. *gambiae* to treated nylon strips

This study was designed to establish whether the physical presence of bacteria on autoclaved IB1-treated and control nylon strips would affect the attraction of host-seeking laboratory-reared *An*. *gambiae* mosquitoes. The study was achieved through randomized dual-choice bioassays comprising (a) non-autoclaved control versus autoclaved control nylon strips, (b) non-autoclaved control versus autoclaved IB1-treated nylon strips, (c) non-autoclaved control versus non-autoclaved IB1-treated nylon strips, (d) autoclaved control versus autoclaved IB1-treated nylon strips, (e) autoclaved control versus non-autoclaved nylon strips, and (f) non-autoclaved control versus autoclaved IB1-treated nylon strips. The experiments were randomized by treatment and trap location. Nylon strips used had been exposed previously for attraction of mosquitoes at weekly intervals for a period of 52 weeks post-treatment in a semi-field environment. Each bioassay was conducted for four nights inside a screen-walled greenhouse. The autoclaved and non-autoclaved IB1-treated and control nylon strips for individual treatments were separately wrapped in aluminium foil and refrigerated at -4°C between experimental nights. During autoclaving, control and IB1-treated nylon strips were separately placed in a 200 ml glass bottle (Pyrex, England), sealed and autoclaved to 121°C at100 kPa (15 psi) for 30 min (Webeco Vertikal model B-C-H-stand, Germany) prior to the start of each experiment. Non-autoclaved treatments were retained at 4°C between experimental nights. Treatments were alternated between trap positions and used repeatedly over the entire study period.

The same treatments used to investigate the effect of autoclaving IBI-treated nylon strips on mosquito attraction under semi-field conditions were also tested outdoors (18:30–06:30 h) at Kigoche village for 20 consecutive nights (December 2011 to January 2012) without re-treatment. This field study was evaluated through a 4 × 4 Latin square experimental design randomized by treatment and house location. Kigoche village is situated near Ahero town in the Kano plains of Kisumu County, western Kenya [23]. Individual treatments assigned to specified MM-X traps were suspended outdoors under the eave adjacent to the bedroom of selected houses. The treatments were alternated at nightly intervals among the different experimental houses. At the end of each experimental night, adult mosquitoes caught in each trap were collected and transported to a field laboratory located at the Ahero Multipurpose Development Training Institute (AMDTI) for sorting and counting. Mosquitoes collected from each trap were killed by freezing, identified morphologically [32,33], counted and recorded based on sex and genus or species (*An*. *gambiae* sensu lato, *An*. *funestus*, *Culex* species, *Mansonia* species, and other anophelines). Other anophelines refer to trapped species of *Anopheles* other than *An*. *gambiae* s.l. and *An*. *funestus*.

### Ethical approval

This study was approved by the ethical review committee of the Kenya Medical Research Institute (KEMRI ERC NON-SSC No. 350). In all cases, the purpose and procedures of the study were explained to local leaders, household heads and volunteers before seeking for permission to carry out the study. Experiments were only done in local houses whose owners consented after having read and understood the protocol of the study prior to signing two copies of the written consent form approved by the ethics committee of KEMRI. One of the copies was kept by the participant while the second one was retained by the project.

### Data analysis

A baseline-category logit model was used to analyse residual activity of blend IB1 on attraction of *An*. *gambiae* mosquitoes [34], while a χ^2^-test was used to analyse for each two-choice test conducted in the olfactometer and screen-walled green house. The effect of autoclaving on attractiveness of IB1-impregnated nylon strips to field mosquitoes trapped during the randomised 4 × 4 Latin square experimental design by treatment and house location was evaluated by using a Generalized Linear Model (GLM) fitted with Poisson distribution and a logarithm link function [35,36]. The effects of treatment and house position on mosquito catches were tested as parameters in the model. The GLM was followed by pairwise comparisons with Least Square Difference correction to test for differences in trap entry response between treatments. All analyses were performed using IBM SPSS statistical software, version 20.0.

## Results

### Long-lasting attraction of *An*. *gambiae* to treated nylon under semi-field conditions

**Fig 1:**
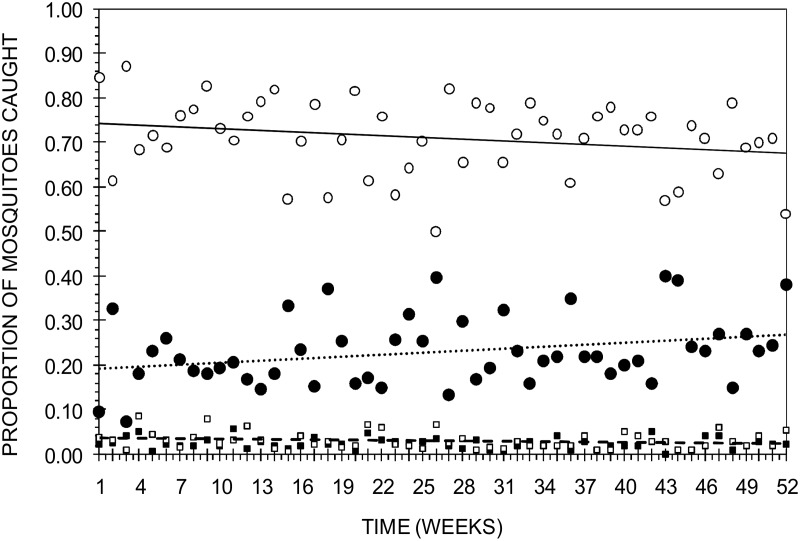
Proportions of female *An*. *gambiae* mosquitoes caught weekly for one year under semi-field conditions in traps containing IB1-treated nylon strips (○), LDPE sachets filled with the IB1 odour blend (●), control nylon strips (□) and control LDPE sachets (■). The treatments were used repeatedly for 52 nights without refreshing them. The solid, dotted, dashed and dashed-with-square lines represent the baseline-category logit model fit showing trends of proportions of mosquitoes attracted over time.

A total of 15,272 female *An*. *gambiae* mosquitoes were caught in all traps used over the one year study period. The mosquitoes were found in traps containing control LDPE sachets (n = 397; 2.6%), control nylon strips (n = 428; 2.8%), LDPE-sachets filled with IB1 (n = 3,344; 21.9%) and IB1-treated nylon strips (n = 11,103; 72.7%) (Fig. 1). Significant and consistently higher proportions of mosquitoes were attracted to IB1-treated nylon strips than the other treatments over the one year study period (P < 0.001). Control LDPE sachets and control nylon strips attracted similar proportions of mosquitoes over time (P = 0.20).

### Identification of compounds found on attractant-treated nylon strips

GC-MS analysis of the IB1-treated nylon strips revealed 28 volatile organic compounds (Table 1). Pentanoic acid and 3-methyl butanoic acid were the only compounds detected from the original IB1 constituents impregnating the strips. The most abundant volatiles were pentanoic acid, 2-(2-methoxyethoxy) ethanol, 2-methoxyethanol, 3-methylbutanoic acid, benzyl alcohol, ethyl lactate, styrene, dihydromyrcenol, nonanal, butyl heptanoate and 1-methylene-1H-indene.

**Table 1:** Volatile compounds found on IB1-impregnated nylon strips after 52 nights of repeated post-treatment exposures at weekly intervals for the attraction of female *An*. *gambiae* mosquitoes under semi-field conditions.

Compound	CAS no	LRI_exp._	LRI_lit._	Identification	Peak Areas
**Alcohols**					
2-Methoxyethanol	109-86-4	627	624	LRI, MS	315528574
1-Butanol	71-36-3	655	655	LRI, MS	35270870
3-Methyl-1-butanol	123-51-3	729	731	LRI, MS	2949860
1-Pentanol	71-41-0	764	766	LRI, MS	4931440
2-(2-Methoxyethoxy) ethanol	111-77-3	937	932	LRI, MS	423644711
2-Ethyl-1-hexanol	104-76-7	1029	1029	LRI, MS	10723899
Benzyl alcohol	100-51-6	1042	1042	LRI, MS	138027214
**Terpenoids**					
Alpha-pinene	80-56-8	946	942	LRI, MS	11108033
Dihydromyrcenol	18479-58-8	1074	1074	LRI, MS	69267580
Isopulegol	89-79-2	1164	1150	LRI, MS	6897405
Neoiso-pulegol	21290-09-5	1177	1171	LRI, MS	11155373
Camphor	76-22-2	1169	1170	LRI, MS	2013866
Citronellyl formate	105-85-1	1231	1249	LRI, MS	817694
**Esters**					
Methyl methacrylate	80-62-6	707	710	LRI, MS	8679905
Ethyl lactate	97-64-3	813	813	LRI, MS	112953541
Ethyl octanoate	106-32-1	1195	1197	LRI, MS	18129873
Butyl heptanoate	5454-28-4	1288	1291	LRI, MS	61244868
Butyl octanoate	589-75-3	1387	1387	LRI, MS	14932759
**Alkylbenzenes**					
Styrene	100-42-5	899	897	LRI, MS	69404000
Pseudocumene	95-63-6	1005	1016	LRI, MS	16709061
1-Methylene-1H-indene	2471-84-3	1215	NA	MS	56319232
**Carboxylic Acids**					
3-Methylbutanoic acid	503-74-2	837	836	LRI, MS	152155177
Pentanoic acid	109-52-4	879	881	LRI, MS	450075877
**Aldehydes**					
(*E*)-2-Methyl-2-butenal	497-03-0	741	739	LRI, MS	3734889
Nonanal	124-19-6	1107	1103	LRI, MS	66692533
**Alkane**					
3-Methyloctane	2216-33-3	873	870	LRI, MS	4514113
**Ketone**					
3-Penten-2-one	625-33-2	735	735	LRI, MS	5318360
**Furan**					
2,3-Diethyl-4,5-dimethylfuran	54244-89-2	1239	NA	MS	654791

The compounds were tentatively identified based on linear retention indices (LRI) and/or mass spectra (MS).

CAS no: refers to a standard identification number of the compound.

LRI_exp_: linear retention indices experimentally obtained.

LRI_lit_: linear retention indices obtained from literature [NIST 2005 and Wageningen University Mass Spectral library) on a column with (5%-Phenyl)-methylpolysiloxane stationary phase or equivalent.

Identification (tentatively) based on retention indices (LRI) and/or mass spectra (MS).

Average peak areas obtained in the samples.

**NA**: Not Available.

### Identification of bacteria found on attractant-treated nylon strips

**Fig 2:**
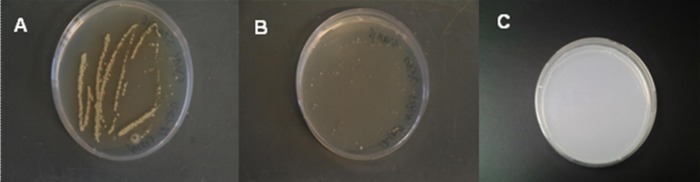
Bacterial culture plates showing results of streaking with IB1-treated nylon strips used repeatedly for collecting female *An*. *gambiae* mosquitoes (one night per week) for one year (panel A), strips of a nylon sock worn for 12 h by a human volunteer (panel B) and control (untreated) nylon strips (panel C).

Plating IB1-treated nylon strips on TSA resulted in more bacterial growth than plating a worn nylon sock on the same agar. Very few bacterial colonies were observed on agar plates streaked with control nylon strips. Sequencing of the bacterial cultures showed that, although many bacterial populations were present on the plate streaked with IB1-treated nylon strips (Fig. 2), *Bacillus thuringiensis* IBL 4222 contig00573 and *Acinetobacter baumannii* ABNIH4 contig00148 were the most abundant.

### Effect of bacteria-produced volatiles on attraction of *An*. *gambiae* in the laboratory

**Fig 3:**
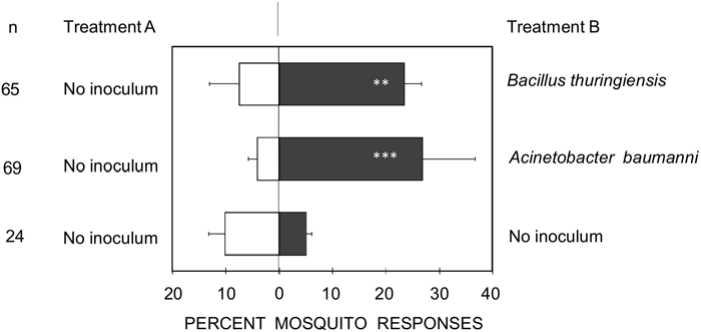
Percent of female *Anopheles gambiae* mosquitoes choosing either treatment A or B in a dual choice olfactometer assay. Blends of volatile organic compounds were released from nutrient agar blocks inoculated with *B*. *thuringie*nsis and *A*. *baumannii* isolates or nutrient agar blocks only. Each treatment was tested four times over two days. The total number of mosquitoes released was 120. The total number of mosquitoes trapped per choice test (n) is shown. Error bars represent standard errors of the mean mosquito percentage. Asterisks indicate significant differences between treatment A and B; ** P < 0.01; *** P < 0.001 (χ^2^ test).

Olfactometer traps baited with blocks of TSA containing *B*. *thuringiensis* IBL 4222 contig00573 or *A*. *baumannii* ABNIH4 contig00148 caught significantly more mosquitoes than the traps with agar alone (P = 0.002 and P < 0.001, respectively) (Fig. 3). There was no significant difference between the responses of mosquitoes caught in the left and the right traps of the olfactometer when both contained agar alone (P = 0.16).

### Effect of autoclaving on attraction of *An*. *gambiae* to treated nylon

**Fig 4:**
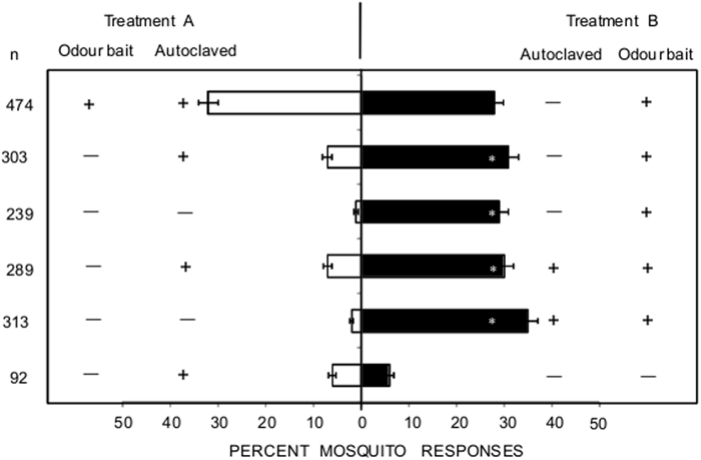
Percent of female *Anopheles gambiae* mosquitoes choosing either treatment A or B in a semi-field dual choice assay. The effect of autoclaving on attraction of female *Anopheles gambiae* mosquitoes to untreated (―) and odour baited (blend IB1) (+) nylon strips was assessed. Nylon strips were either non-autoclaved (―) or autoclaved (+). The total number of mosquitoes released (N) for each dual choice test, replicated during four nights, was 800. The total number of mosquitoes trapped per choice test (n) is shown. Error bars represent standard errors of the mean. Bars followed by * denote significant differences (P < 0.05) in mosquito catches between treatments.

The semi-field assay resulted in a higher attraction of *An*. *gambiae* to non-autoclaved IB1-impregnated nylon strips than to non-autoclaved (P < 0.001) and autoclaved (P < 0.001) control nylon strips (Fig. 4). Similarly, a considerably higher proportion of *An*. *gambiae* responded to autoclaved IB1-treated nylon strips than to non-autoclaved (P < 0.001) and autoclaved (P < 0.001) control nylon strips. However, *An*. *gambiae* mosquitoes responded equally to non-autoclaved versus autoclaved IB1-treated nylon strips (P = 0.17), as well as to non-autoclaved control versus autoclaved control nylon strips (P = 0.68).

Field studies were conducted from December 2011 to January 2012. The average temperature, RH and total rainfall over this period were 24.5 ± 0.8°C, 75.3 ± 9.1%, and 294.7 mm, respectively. A total of 1,547 mosquitoes including 237 (15.3%) males and 1,310 (84.7%) females were trapped. The female mosquitoes comprised *An*. *gambiae* s.l. (8.2%), *An*. *funestus* (16.1%), *Culex* spp. (45.6%), *Mansonia* spp. (3.1%) and other anopheline spp. (dominated by *An*. *coustani* Laveran and *An*. *ziemanni* Grunberg) (27.0%) (Table 2). Autoclaving had no effect on the responses of female *An*. *gambiae* s.l. to IB1-treated nylon strips (P = 0.26). Autoclaved IB1–treated nylon strips were more attractive to female *An*. *funestus* (P < 0.001) and other anopheline mosquitoes (P < 0.007) compared to non-autoclaved IB1-impregnated nylon strips. On the contrary, larger numbers of female *Culex* spp. responded to non-autoclaved IB1-treated nylon strips than to autoclaved IB1-treated nylon strips (P < 0.042). In addition, similar numbers of female *Mansonia* spp. responded to non-autoclaved and autoclaved IB1-treated nylon strips (P = 0.17).

**Table 2:** Mean number (±SE) of female mosquitoes caught outdoors in traps containing autoclaved and non-autoclaved control nylon strips (without odor) or IB1-treated nylon strips at Kigoche village in western Kenya.

Treatment	N	Mean number ± SE of mosquitoes caught/trap/night				
		*An*. *gambiae* s.l.	*An*. *funestus*	*Culex* spp.	*Mansonia* spp.	Other anophelines
Control non-autoclaved nylon strips	20	0.15 ± 0.09^a^	0.65 ± 0.2^a^	1.68 ± 0.27^a^	0.13 ± 0.06^a^	1.25 ± 0.22^a^
Control autoclaved nylon strips	20	0.60 ± 0.17^a^	0.75 ± 0.19^a^	3.20 ± 0.39^b^	0.28 ± 0.12^a^	1.18 ± 0.09^a^
Non-autoclaved IB1-treated nylon strips	20	2.05 ± 0.32^b^	3.30 ± 0.41^b^	12.07 ± 0.77^c^	0.56 ± 0.16^b^	5.57 ± 0.52^b^
Autoclaved IB1-treated nylon strips	20	2.60 ± 0.36^b^	5.85 ± 0.54^c^	10.03 ± 0.70^d^	0.92 ± 0.22^b^	7.56 ± 0.63^c^

The number of experimental nights (N) and standard error of the mean mosquito catches per night (SE) are shown. Mean values within the same column with different superscript letters differ significantly (P < 0.05, GLM).

The 237 male mosquitoes caught comprised *An*. *gambiae* s.l. (26.2%), *An*. *funestus* (36.3%), *Culex* spp. (24.9%), *Mansonia* spp. (4.2%) and other Anopheline spp. (8.4%) (Table 3). Trap catches of male *An*. *gambiae* s.l., *An*. *funestus* and *Culex* spp. were dependent on treatment type (P < 0.001 for all). Significantly more males of *An*. *gambiae* s.l. (P < 0.022), *An*. *funestus* (P < 0.004) and *Culex* spp. (P < 0.001) mosquitoes were attracted to autoclaved IB1-treated nylon strips compared to non-autoclaved IB1-treated nylon strips. The mean numbers of male *Mansonia* spp. and other anopheline mosquitoes collected between IB1-baited traps were not different (P = 0.14 and P = 1.00, respectively).

**Table 3:** Mean number (±SE) of male mosquitoes caught outdoors in traps containing autoclaved and non-autoclaved control nylon strips or IB1-treated nylon strips at Kigoche village in western Kenya.

Treatment	N	Mean number ± SE of mosquitoes caught/trap/night				
		*An*. *gambiae* s.l.	*An*. *funestus*	*Culex* spp.	*Mansonia* spp.	Other anophelines
Control non-autoclaved nylon strips	20	0.25 ± 0.11^a^	0.35 ± 0.13^a^	0.65 ± 0.18^a^	0.10 ± 0.07^a^	0.15 ± 0.09^a^
Control autoclaved nylon strips	20	0.20 ± 0.10^a^	0.55 ± 0.17^a^	0.85 ± 0.21^a^	0.10 ± 0.07^a^	0.05 ± 0.05^a^
Non-autoclaved IB1-treated nylon strips	20	0.90 ± 0.21^b^	1.10 ± 0.23^b^	0.25 ± 0.11^b^	0.05 ± 0.05^a^	0.40 ± 0.14^a^
Autoclaved IB1-treated nylon strips	20	1.75 ± 0.30^c^	2.30 ± 0.34^c^	1.20 ± 0.24^a^	0.25 ± 0.11^a^	0.40 ± 0.14^a^

The number of experimental nights (N) and standard error of the mean mosquito catches per night (SE) are shown. Mean values within the same column with different superscript letters differ significantly (P < 0.05).

## Discussion

This study indicates that consistently more female *An*. *gambiae* mosquitoes were attracted to IBI dispensed from nylon strips than to LDPE sachets or untreated controls tested at weekly intervals up to one year post-treatment. After one year of repeated use, IB1-treated nylon strips released compounds that were absent from the initial blend applied. Of the cultured bacteria isolated from such strips, the most abundant species were *B*. *thuringiensis* and *A*. *baumannii*. Volatile compounds released by these two bacteria attracted significantly more female *An*. *gambiae* mosquitoes than agar alone in the olfactometer. Follow-up experiments demonstrated that both autoclaved and non-autoclaved IB1-treated nylon strips were equally attractive to female *An*. *gambiae* in a semi-field assay and also to wild female *An*. *gambiae* s.l. and *Mansonia* spp. under field conditions. However, whereas autoclaving enhanced the attractiveness of IB1-treated nylon strips to female *An*. *funestus* and other anopheline mosquitoes, the majority of female *Culex* spp. were by contrast attracted to non-autoclaved IB1-treated nylon strips as opposed to the autoclaved IB1-treated comparators.

These findings confirm that nylon strips provide a better matrix for dispensing attractant compounds than LDPE sachets [14,15]. A recent semi-field study showed that IB1-treated nylon strips were similarly more attractive to host-seeking mosquitoes than LDPE sachets filled with IB1 up to 40 consecutive nights post-treatment [15]. The current study corroborates and extends these findings up to a one year post-treatment period. The prolonged residual activity of attractant-treated nylon strips points towards the possibility and robustness of using odor-baited tools for field operations. These findings agree with the long-term attraction of female *An*. *gambiae* mosquitoes to human emanations collected on nylon and cotton materials reported a decade ago [17,18].

Investigations of whether the original chemicals impregnated on nylon strips were still present confirmed pentanoic acid and 3-methyl butanoic acid as the only compounds left one year post-treatment. Twenty six additional compounds were identified. Of the 26 compounds, 1-butanol, 3-methylbutanoic acid and 3-methyl-1-butanol are known to be produced by skin bacteria or found in skin sweat [19,20,37], while (*E*)-2-methyl-2-butenal, pentanoic acid and styrene are produced in human skin [38,39]. Whereas 1-pentanol is found in human sweat and also produced by yeast [37], benzyl alcohol is a constituent of hand odor as well as a product of bacterial activity [40]. These observations suggest that IB1-treated nylon strips may have been colonised by bacteria, which contributed to the catabolism and release of additional chemicals [20,41,42]. For example, 1-butanol and 3-methyl-1-butanol are bacterial break-down products of tetradecanoic acid on human skin [19,20]. Indeed, a novel synthetic odor blend of 3-methyl-1-butanol, L-lactic acid, ammonia and tetradecanoic acid has been shown to be more attractive to host-seeking *An*. *gambiae* s.l. and *An*. *funestus* compared to blend IB1 under semi-field and field conditions [23,34]. Furthermore, nonanal detected in the headspace of IB1-treated nylon strips is one of the human-derived volatile compounds that play an integral role in differential attractiveness of human volunteers to mosquito vectors [43]. Individuals that release higher quantities of three aldehydes (octanal, nonanal and decanal) and two ketones (geranylacetone and 6-methyl-5-hepten-2-one) have been shown to be ‘unattractive’ to *Aedes aegypti* Linnaeus and *An*. *gambiae* mosquitoes while those emitting lower quantities of the same compounds are ‘attractive’ [4,43].

Host-seeking behavior of *An*. *gambiae* and other dipterans is mediated by VOCs produced from either skin glands or skin microflora, or both [44,45]. The role of bacterial volatiles in modulating mosquito host-seeking behavior has been demonstrated [19,20]. Subsequent evidence has shown that VOCs produced by skin microbiota affect differential attractiveness of humans through stimulation or inhibition of host-seeking responses of mosquito vectors although some are neutral [13,20]. For example, the attractiveness of a simple odor blend containing ammonia, L-lactic acid and tetradecanoic acid to *An*. *gambiae* mosquitoes was reduced by addition of 2-phenylethanol but enhanced when combined with 3-methyl-1-butanol [13]. Thus, the association between human skin microbiota and production of odorous compounds that function as attractants for host-seeking mosquitoes can be exploited for monitoring malaria vectors [19,20,36].

It has also been proposed that human attractiveness to *An*. *gambiae* and other mosquitoes is affected by species composition, density, and metabolic activity of the skin microbiota [19,20]. However, there is no information about the type and role of bacteria that colonize attractant-treated nylon strips. Indeed, this potential was demonstrated by the higher attraction of female *An*. *gambiae* mosquitoes to volatiles produced by *B*. *thuringiensis* and *A*. *baumannii* bacteria than to agar alone in the olfactometer. The present findings suggest that residual attractiveness of IB1-treated nylon strips over time is likely to be affected by bacteria that establish on strips over time and could be partly ascribed to the emission of additional volatiles. Plating of IB1-treated nylon strips resulted in more bacterial growth than plating of a nylon sock that had been worn for only 12 h or untreated nylon strips, possibly because treated nylon strips had been exposed for a longer time (i.e. 52 nights) and this is likely to have increased bacterial activity [16]. Furthermore, not all cultivatable bacteria may have grown probably because incubation overnight was a short time period for the growth of all cultivatable bacteria.

Follow-up experiments demonstrated that autoclaving of IB1-treated nylon strips prior to deployment did not cause any appreciable effect on their attractiveness to host-seeking *An*. *gambiae* s.l. and *Mansonia* mosquitoes. However, the effect was more profound on wild female *An*. *funestus*, *Culex* spp., and other anophelines including wild male *An*. *gambiae* s.l., *An*. *funestus* and *Culex* spp each of which was more attracted to autoclaved than non-autoclaved IB1-treated nylon strips. In general and contrary to our expectations, autoclaving did not abolish the attraction of host-seeking mosquitoes to IB1-treated nylon strips. This implies that the physical presence of live bacteria is not required for attraction of mosquitoes to odor baits or hosts. Rather attraction of host-seeking mosquitoes is mediated by chemicals emanating from bacterial metabolism. The results suggest that upon deployment, bacteria may have colonised IB1-treated nylon strips and produced compounds attractive, repellent or neutral to mosquitoes, thereby changing the composition of the blend or nylon. Subsequently, upon autoclaving, bacteria were killed but VOCs already produced persisted or the bacteria recolonised the strips rapidly. However, most notable is the fact that bacterial spores are not destroyed through autoclaving [46]. Alternatively, heating may have released attractive volatiles from the bacterial cells, helped to volatilize attractant compounds or changed their composition on the nylon and/or synthetic components. It is also possible that in spite of continued loss of volatile compounds from treated nylon strips over time due to repeated exposure or autoclaving, mosquitoes still had the capacity to respond to traces of the original compounds that were left behind. Perhaps, traces of original compounds found on treated nylon strips were below the detection limit of GC-MS protocol used in the current study. On these bases, autoclaving is likely to have influenced the composition and concentration of the odor plume encountered by the mosquitoes. These tentative hypotheses require evidence-based explanations hence the need for additional research on the dynamics of bacterial communities and composition of volatiles released on odor dispensing nylon. The type of volatiles emitted from the control TSA and bacterial culture on TSA should also be identified.

The field conditions that prevailed seem to have favoured a higher abundance of *An*. *funestus* compared to *An*. *gambiae* s.l. (which were identified to 100% *An*. *arabiensis*) as the studies were conducted in a dry season and they also coincided with maturation of rice grown in adjacent paddies [47,48]. This is because *An*. *funestus* breeds in slow-moving water containing emergent vegetation whereas *An*. *gambiae* and *An*. *arabiensis* prefer temporary, shallow and sunlit water bodies [48]. Similar to previous studies, the odor bait used was more selective for female than male mosquito populations because only females engage in blood feeding [12,14,21]. The odor blend was also associated with human host odorants to which host-seeking female mosquitoes respond. Thus, the blend may be deployed effectively for sampling, surveillance as well as intervention of mosquito vectors [7,49].

## Conclusion

This study has demonstrated the feasibility of using synthetic odor baits for sampling and surveillance of malaria and other mosquito vectors over prolonged periods of time, during which the baits did not lose attraction to mosquitoes. Preliminary evidence points towards the potential role of bacteria in sustaining prolonged use of nylon material for dispensing synthetic attractant odorants for host-seeking malaria and other mosquito vectors but further investigations are required.
